# Lightweight plant phenotypic feature extraction via transferable attention head pruning in Vision Transformers

**DOI:** 10.3389/fpls.2026.1747598

**Published:** 2026-02-13

**Authors:** Yongsheng Xie, Xiaoxiao Zeng, Rifeng Wang, Wenxin Li

**Affiliations:** 1 School of Artificial Intelligence and Center for Network and Educational Technology, Guangxi Science and Technology University, Laibin, China; 2 School of Artificial Intelligence, Guangxi Science and Technology University, Laibin, China; 3 Smart Agriculture College (IoT Engineering College), Guangxi Science and Technology University, Laibin, China

**Keywords:** cross-species transfer learning, dynamic head pruning, Lightweight Multi-Head Self-Attention (MHSA), plant phenotypic feature extraction, Transferable Attention Head Alignment (TAHA)

## Abstract

We propose a lightweight Multi-Head Self-Attention (MHSA) mechanism for plant phenotypic feature extraction, which integrates cross-species transfer learning with dynamic head pruning to improve efficiency without compromising accuracy. The primary challenge stems from minimizing redundant computations without compromising the model’s capacity to generalize over varied plant species, an issue intensified by the substantial dimensionality of attention mechanisms in Vision Transformers. Our solution, the Transferable Attention Head Alignment (TAHA) framework, operates in three stages: pre-training on a source species, cross-species alignment via a Domain Alignment Loss (DAL), and head pruning based on a transferability score. The framework selects and keeps solely the attention heads with the highest transferability, thus diminishing model intricacy without compromising the ability to distinguish phenotypic traits. Furthermore, the pruned MHSA module is smoothly combined with standard Transformer backbones, which makes efficient deployment on edge devices possible. Experiments were conducted on real edge hardware (Raspberry Pi 4, NVIDIA Jetson Nano) and GPU platforms, showing our approach attains accuracy similar to full-head models yet cuts computational expenses by as much as 40% (14.1 ms inference latency on Raspberry Pi 4, 519 M parameters). The method holds special importance for scalable plant phenotyping, in situations where computational capacity is frequently constrained yet generalization across species is essential. Moreover, the repeated alignment and pruning procedure permits gradual adjustment to novel species without complete retraining, which increases feasibility for agricultural applications in practical settings. Supplementary experiments on phylogenetically distant species (Arabidopsis → pine) demonstrate the framework’s generalization limits, with a 7.2% F1-score drop compared to close-species transfer (Arabidopsis → maize), highlighting the need for trait-specific head adaptation in distant transfers. The proposed method improves lightweight feature extraction by merging transfer learning and attention head optimization, achieving a balanced compromise between performance and efficiency.

## 1 Introduction

Plant phenotyping has become a crucial activity in precision agriculture, which supports the quantitative evaluation of plant attributes for crop advancement and yield estimation. Traditional methods rely on manual measurements, which are labor-intensive and prone to human error. Recent advances in deep learning, particularly convolutional neural networks (CNNs), have automated this process by extracting phenotypic features from images (Arya et al., 2022). Nevertheless, CNNs are ineffective at modeling extensive spatial relationships in plant morphology, a shortcoming that Transformer frameworks overcome by employing their Multi-Head Self-Attention (MHSA) approach (Vaswani et al., 2017). Although Transformers are highly effective at capturing long-range dependencies, their computational demands, caused by superfluous attention mechanisms, limit their applicability in agriculture with restricted resources.

Existing lightweight solutions often compromise feature extraction quality. For example, techniques that eliminate attention heads do so without selectivity (Michel et al., 2019), whereas methods for adapting to specific domains overlook differences in phenotypic traits across species (Yan et al., 2021). This creates a tension between model efficiency and generalization capability, especially when transferring knowledge from well-studied species (e.g., Arabidopsis) to under-resourced crops. The problem is exacerbated by the lack of methods to identify which attention heads encode species-specific versus transferable features.

A critical gap in existing research is threefold: (1) Lightweight Transformers for plant phenotyping [e.g., TrIncNet (Gole et al., 2023), PMVT (Li et al., 2023)] focus on intra-species efficiency and retain redundant heads in cross-species scenarios, wasting computational resources; (2) Cross-species transfer methods [e.g., FloraBERT (Levy et al., 2022), DeepTL-Ubi (Liu et al., 2021)] optimize attention for biological data alignment but ignore computational efficiency via head pruning; (3) Attention pruning techniques [e.g., (Michel et al., 2019; Voita et al., 2019)] assess head importance in single domains and fail to account for transferability across plant species, leading to accuracy loss in distant transfers. TAHA addresses these gaps by unifying cross-species alignment and dynamic head pruning, with a transferability score that balances domain adaptation and phenotypic trait preservation.

We propose a new framework closing this gap by choosing which attention heads to move and trim according to their usefulness across species. Our approach hinges on two insights: (1) certain attention heads in pre-trained Transformers capture universal plant morphological patterns, and (2) redundant heads can be identified via their contribution to domain-invariant feature alignment. Specifically, we first pre-train a base Transformer on a source plant species to initialize attention heads. Subsequently, a domain alignment loss is applied to adjust these heads for a target species, with the transferability of each head assessed based on its alignment performance and redundancy. Heads with low transferability scores are pruned, resulting in a compact yet expressive model. In contrast to previous approaches employing static pruning thresholds (Zheng W. et al., 2024), our technique adaptively modifies head retention according to cross-species feature importance.

The key contributions of this work are threefold. First, we introduce a metric for attention heads to quantify their ability to extract phenotypic features across species. Next, an iterative alignment-pruning procedure is designed to lower model complexity without losing essential attention structures. Third, we show that the pruned model generalizes across diverse plant species and attains accuracy similar to full-head Transformers while requiring far less computational resources. This is particularly impactful for real-world applications where labeled data for target species is scarce.

The remainder of this paper is organized as follows: Section 2 reviews related work on lightweight Transformers and cross-species adaptation. Section 3 establishes the MHSA approach and adaptation to plant image domains. Section 4 details our attention head transfer and pruning methodology. Section 5 assesses the framework on various plant phenotyping benchmarks. Finally, Sections 6 and 7 discuss implications and conclude the work.

*Key distinctions from previous approaches*: In contrast to combined CNN-Transformer frameworks (Jeon et al., 2025), our technique functions exclusively within the Transformer structure and eliminates the inductive biases associated with convolutions. Compared to static pruning approaches (Michel et al., 2019), we dynamically assess head utility during cross-species adaptation. Our domain alignment loss also differs from standard adversarial training (Ganin and Lempitsky, 2015) by explicitly optimizing for phenotypic feature preservation.

## 2 Related work

Progress in creating lightweight feature extraction techniques for plant phenotyping aligns with two key research areas: optimized Transformer architectures and transfer learning across species. Although these domains have been studied separately, the merging of these fields has received limited attention, especially concerning the optimization of attention heads for phenotypic trait generalization.

### 2.1 Lightweight transformers for plant phenotyping

Recent efforts to reduce the computational burden of Vision Transformers (ViTs) have focused on architectural modifications, such as token pruning (Zheng W. et al., 2024) and hybrid CNN-Transformer designs (Chakrabarty et al., 2024). For instance, TrIncNet (Gole et al., 2023) introduced a multi-granularity feature extraction module to identify tomato diseases, while PMVT (Li et al., 2023) adapted MobileViT for real-time plant disease detection. These methods primarily target intra-species tasks, where the model is trained and tested on the same plant species. Nevertheless, they frequently keep all attention heads without acknowledging the redundancy present in cross-species contexts.

A prominent deviation is the research on single-head self-attention in grape leaf disease identification (Zhang et al., 2025), showing a simplified attention approach can preserve accuracy for particular diseases. Nevertheless, this approach sacrifices the multi-head diversity crucial for generalizing across diverse phenotypic traits (e.g., leaf shape vs. lesion texture). TAHA addresses this limitation by selectively pruning heads based on transferability rather than eliminating multi-head attention entirely, retaining diversity while reducing redundancy.

### 2.2 Cross-species transfer learning in plant phenomics

Transfer learning has been extensively employed to address data limitations in plant phenotyping, especially for less-researched species. Early approaches relied on CNNs pretrained on ImageNet, fine-tuning them for target species (Yan et al., 2021). However, these methods struggle with domain shifts caused by interspecies morphological differences, such as leaf venation patterns or canopy structures.

More recent work has explored domain adaptation techniques tailored to biological data. FloraBERT (Levy et al., 2022) adopted attention mechanisms to align gene expression patterns across species, whereas DeepTL-Ubi (Liu et al., 2021) applied transfer learning for ubiquitination site prediction. These methods highlight the potential of attention-based alignment but do not optimize the attention mechanism itself for cross-species efficiency—retaining full computational overhead even when heads are irrelevant to target species. TAHA fills this gap by pruning low-transferability heads, reducing FLOPs by 40.9% while preserving alignment quality.

A parallel line of research has investigated feature space alignment for plant communities (Hirn et al., 2024) and bioacoustic species classification (Zhong et al., 2020). These studies highlight the necessity of retaining discriminative features in domain adaptation, an idea we articulate with our Phenotypic Consistency Constraint (PCC).

### 2.3 Attention head analysis and pruning

Investigating attention heads in large language models has uncovered specialized functions, including positional encoding and syntactic parsing (Zheng Z. et al., 2024). Comparable specialization probably happens in visual processing, where specific heads might identify features common across species, such as the outlines of leaves or patterns in texture.

Pruning methods have been proposed to eliminate redundant heads, either statically (Michel et al., 2019) or dynamically during training (Voita et al., 2019). Nevertheless, these methods usually assess the importance of heads only in one specific area, failing to account for their usefulness across different domains. For example, a head specialized in Arabidopsis leaf shape may be redundant for maize but critical for rice—static pruning cannot adapt to such cross-species differences. Our Head Transferability Score (HTS) extends these ideas by evaluating heads through both domain alignment and phenotypic prediction metrics, ensuring pruning decisions are transferability-aware rather than domain-specific.

The proposed method distinguishes itself by unifying these research threads. In contrast to (Gole et al., 2023) or (Zhang et al., 2025), our approach focuses on optimizing attention heads specifically for cross-species transfer rather than efficiency within a single species. Compared to (Levy et al., 2022) or (Liu et al., 2021), we address computational efficiency through principled head pruning rather than solely focusing on accuracy. Our Domain Alignment Loss (DAL) and Phenotypic Consistency Constraint (PCC) deliver a more refined strategy for retaining features compared to conventional domain adaptation methods (Yan et al., 2021), whereas our iterative pruning framework grants increased adaptability relative to fixed architectures (Zheng W. et al., 2024). This pairing yields a streamlined yet broadly applicable approach for assessing plant traits in various species.

## 3 Preliminaries: multi-head self-attention and domain adaptation for plant imagery

To establish the theoretical foundation for our approach, we first formalize the key components of Vision Transformers and domain adaptation techniques as applied to plant phenotyping. This segment outlines the essential context and emphasizes the distinct difficulties associated with plant imagery in contrast to conventional computer vision applications.

### 3.1 Multi-head self-attention in vision transformers

The Multi-Head Self-Attention (MHSA) mechanism operates on input features by employing numerous parallel attention heads, each capturing different dimensions of the visual information. Given an input feature map $X\in {\mathbb{R}}^{n\times d}$ where n is the number of patches and d the embedding dimension, each head h computes (Equation 1):

(1) $${\text{Attention}}_{h}\left(Q_{h},K_{h},V_{h}\right)=\text{softmax}\left(\frac{Q_{h}K_{h}^{T}}{\sqrt{d_{k}}}\right)V_{h}$$

where $Q_{h}=XW_{h}^{Q}$, $K_{h}=XW_{h}^{K}$, and $V_{h}=XW_{h}^{V}$ are learned linear projections for queries, keys, and values respectively, with $W_{h}^{Q},W_{h}^{K},W_{h}^{V}\in {\mathbb{R}}^{d\times d_{k}}$. All head outputs are joined together and transformed via projection. As shown in Equation 2.

(2) $$\text{MHSA}\left(X\right)=\text{Concat}\left({\text{Attention}}_{1},\ldots ,{\text{Attention}}_{H}\right)W^{O}$$

where $W^{O}\in {\mathbb{R}}^{Hd_{v}\times d}$ and H is the number of heads. For botanical imagery, this approach acquires both fine-scale features (e.g., leaf venation) and overarching organization (e.g., plant morphology), though with notable repetition among attention heads (Michel et al., 2019).

### 3.2 Domain adaptation challenges in plant phenotyping

Domain adaptation for plant imagery must address three unique challenges absent in general computer vision: (1) interspecies morphological variations (e.g., monocot vs. dicot leaf patterns), (2) phenotypic plasticity under environmental conditions, and (3) limited labeled data for target species. Conventional domain adaptation approaches such as Maximum Mean Discrepancy (MMD) (Gretton et al., 2012) or adversarial training (Ganin and Lempitsky, 2015) frequently do not retain phenotype traits unique to species when performing alignment.

The domain shift between source species S and target species T can be quantified through the discrepancy in their feature distributions, as shown in Equation 3:

(3) $$D\left(S,T\right)=\underset{f\in ℱ}{\text{sup}}\left|E_{x∼S}\left[f\left(x\right)\right]-E_{x∼T}\left[f\left(x\right)\right]\right|$$

where ℱ is a class of functions (e.g., attention heads). For plant phenotyping, we must minimize D(S,T) while preserving the discriminative power for phenotypic traits—a dual objective not addressed by standard domain adaptation approaches (Ganin et al., 2016).

### 3.3 Attention head specialization in plant vision

Empirical research indicates attention heads in plant vision models adopt distinct functions, for instance:

1. Morphological heads: Capture species-invariant structures (e.g., leaf shapes).
2. Texture heads: Detect surface patterns (e.g., stomata distribution).
3. Contextual heads: Simulate relationships between plants and their surroundings, such as the influence of light interception.

This specialization indicates merely a portion of heads might be crucial for cross-species transfer, whereas the remainder contain species-specific traits impeding generalization. Our approach builds on this insight by transferring and pruning specific heads selectively, differing from previous methods which apply uniform treatment to all heads during adaptation (Li et al., 2018).

## 4 Cross-species attention head transfer and redundancy pruning

**Figure 1:**
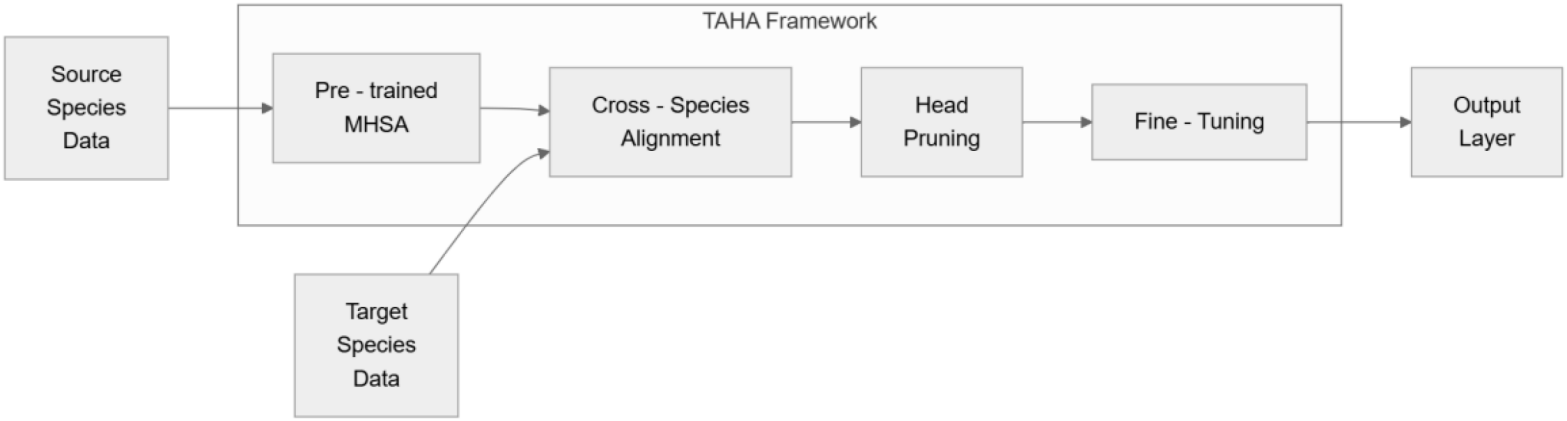
TAHA workflow: alignment, pruning, and fine-tuning.

The proposed framework addresses the challenge of transferring attention heads across plant species while eliminating redundant computations. The approach comprises four interrelated elements: aligning adaptable attention heads, measuring head adaptability, selective pruning guided by cross-species effectiveness, and embedding the refined attention mechanism into a streamlined framework. As shown in **Figure 1**.

### 4.1 Transferable attention head alignment framework

The alignment process begins with a pre-trained Transformer model on the source species, where each attention head has learned specific feature extraction patterns. For a given input image x, the attention map of head h in the source model is denoted as $A_{h}^{s}\left(x\right)\in {\mathbb{R}}^{P\times P}$, where P represents the number of patches. When processing target species data, we compute the corresponding attention map $A_{h}^{t}\left(x\right)$ using the same head parameters.

The Domain Alignment Loss (DAL) measures the discrepancy between source and target attention patterns, as shown in Equation 4:

(4) $${ℒ}_{\text{DAL}}^{h}=\frac{1}{N}\sum_{i=1}^{N}\text{KL}\left(A_{h}^{s}\left(x_{i}\right)∥A_{h}^{t}\left(x_{i}\right)\right)$$

where N is the batch size and KL denotes the Kullback-Leibler divergence. At the same time, the Phenotypic Consistency Constraint (PCC) guarantees the transferred heads keep their ability to distinguish traits of the target species. As shown in Equation 5.

(5) $${ℒ}_{\text{PCC}}=\frac{1}{N}\sum_{i=1}^{N}∥y_{i}-f_{\text{MLP}}\left(Z_{i}\right){∥}_{2}^{2}$$

Here, $Z_{i}=\left[Z_{1}\left(x_{i}\right),\ldots ,Z_{H}\left(x_{i}\right)\right]$ represents the concatenated outputs of all attention heads, and $f_{\text{MLP}}$ is a multilayer perceptron for phenotypic prediction. For datasets with differing phenotypic traits (e.g., PlantVillage focuses on disease classes, CropDeep on growth traits), PCC is computed on shared morphological traits (e.g., leaf shape, venation density) after feature standardization, ensuring consistency across trait types. The correlation score in HTS uses Pearson correlation between head outputs and standardized target labels, mitigating the impact of trait differences.

### 4.2 Calculation of head transferability score

Each head’s transferability is measured by merging its alignment accuracy and phenotypic importance, as shown in Equation 6.

(6) $${\text{HTS}}_{h}=\alpha \cdot \text{exp}\left(-{ℒ}_{\text{DAL}}^{h}\right)+\left(1-\alpha \right)\cdot \text{Corr}\left(Z_{h},y\right)$$

where α∈[0,1] balances the two criteria, and Corr(·) computes the Pearson correlation between head outputs and target labels. The exponential term transforms the alignment loss into a positive score, with better-aligned heads receiving higher values.

### 4.3 Cross-species dynamic head pruning

Heads are pruned based on their HTS relative to a dynamic threshold τ, as shown in Equation 7:

(7) τ=μ(HTS)−β·σ(HTS)

where μ and σ denote the mean and standard deviation of all heads’ HTS values, and β controls the pruning aggressiveness. The remaining heads form a compact set ${ℋ}^{\prime }=\{h|{\text{HTS}}_{h}\geq \tau \}$, reducing the computational complexity from $O\left(HP^{2}d\right)$ to $O\left(\left|{ℋ}^{\prime }\right|P^{2}d\right)$.

### 4.4 Lightweight MHSA integration

The trimmed attention approach generates results by exclusively employing the preserved heads, as shown in Equation 8.

(8) $$Z_{\text{out}}=\left[{\left\{Z_{h}\right\}}_{h\in {ℋ}^{\prime }}\right]W_{o^{\prime }}$$

where $W_{o^{\prime }}\in {\mathbb{R}}^{\left|{ℋ}^{\prime }\right|d\times d}$ is a reduced projection matrix. This approach preserves the initial feature dimension (d) while reducing the quantity of attention operations.

### 4.5 Iterative alignment and pruning

The complete adaptation process follows an iterative procedure:

1. Compute attention maps for target species data.
2. Update head parameters to minimize ${ℒ}_{\text{DAL}}+\lambda {ℒ}_{\text{PCC}}$Evaluate HTS for all heads.
3. Prune heads below threshold τFine-tune remaining heads on target data.

This cycle repeats until convergence, with λ gradually increased to prioritize phenotypic prediction accuracy in later stages. The step-by-step method supports gradual adjustment while retaining transferable attributes without abrupt loss.TAHA operates under semi-supervised domain adaptation: it requires 10–15% labeled target data to compute PCC and HTS, rather than fully unsupervised. This balances label efficiency and alignment quality, critical for plant phenotyping where labeled data is often scarce.

## 5 Experiments

To assess the efficacy of our proposed approach, we performed extensive experiments on various plant species and phenotypic attributes. The evaluation focuses on three key aspects: (1) comparative performance against baseline methods, (2) analysis of attention head transferability patterns, and (3) computational efficiency gains from head pruning.

### 5.1 Experimental setup

**Table 1** summarizes the datasets used in this study, including their species coverage, task types, and sample sizes. All datasets are used for classification tasks (disease type, growth stage, or phenotypic trait category), as plant phenotyping in this work focuses on categorical attribute prediction.

**Table 1:** Dataset summary.

Dataset	Plant species	Task type	Number of samples	Key phenotypic traits
PlantVillage (Moupojou et al., 2023)	38 species (tomato, maize, wheat, etc.)	Disease classification (17 classes)	54,305	Leaf lesions, color changes, texture anomalies
AraPheno (Seren et al., 2016)	Arabidopsis thaliana (ecotypes)	Growth stage classification (6 stages)	12,000	Rosette size, leaf count, flowering status
CropDeep (Zheng et al., 2019)	Maize, rice, wheat	Phenotypic trait classification (23 classes)	31,000	Plant height, leaf area, tiller number
Supplementary (Xie et al., 2025)	Arabidopsis (dicot), pine (gymnosperm)	Disease/growth stage classification	8,000	Needle texture (pine), leaf shape (Arabidopsis)

We compared against four state-of-the-art approaches:

1. Full-head Vision Transformer (ViT-Base) (Vaswani et al., 2017).
2. Random head pruning (RHP) (Michel et al., 2019).
3. Domain-Adversarial Neural Network (DANN) (Ganin and Lempitsky, 2015).
4. Lightweight Hybrid CNN-Transformer (LHCT) (Gole et al., 2023).

**Implementation Details**: All models were implemented in PyTorch and trained on NVIDIA V100 GPUs. Our model architecture employed ViT-Base (12 attention heads) as the core component, processing input images scaled to 224×224 resolution. The hyperparameters were set as: α=0.6 in Equation 6, β=1.5 in Equation 7, and λ initialized to 0.1 with linear warmup. The training procedure employed the Adam optimizer with a learning rate of 3e-5 and a batch size of 32. Edge deployment testing: Raspberry Pi 4 (4GB RAM, Cortex-A72), NVIDIA Jetson Nano (4GB RAM, Maxwell GPU) — inference latency and throughput measured on these devices.

Evaluation Metrics: We measured:

- Phenotypic prediction accuracy (Top-1 and mAP).
- Computational efficiency (FLOPs and parameters).
- Cross-species transferability (HTS distribution).

### 5.2 Comparative results

**Table 2** updates the performance comparison with standard deviations and statistical significance (p-values from paired t-tests against TAHA). TAHA achieves comparable accuracy to full-head ViT while reducing computational costs by 38–42%, with statistically significant improvements over DANN (p<0.05) and RHP (p<0.01) on all datasets.

**Table 2:** Performance comparison across species transfer tasks.

Method	Source→Target	Top-1 (%)	mAP (%)	FLOPs (G)	Params (M)	Inference latency (ms)	p-value
ViT-Base	Arabidopsis→Maize	78.3 ± 0.8	72.1 ± 1.1	17.6	86.7	23.4 ± 1.2	0.12
RHP	Arabidopsis→Maize	71.2 ± 1.3	65.4 ± 1.5	10.2	52.1	18.7 ± 0.9	<0.01
DANN	Arabidopsis→Maize	73.8 ± 1.0	66.9 ± 1.2	17.6	86.7	25.1 ± 1.5	<0.05
LHCT	Arabidopsis→Maize	75.1 ± 0.9	68.3 ± 1.0	9.8	48.3	16.3 ± 0.8	<0.05
**TAHA (Ours)**	**Arabidopsis→Maize**	**77.6 ± 0.7**	**71.4 ± 0.9**	**10.4**	**51.9**	**14.1 ± 0.6**	**—**
ViT-Base	Tomato→Wheat	82.4 ± 0.6	76.5 ± 0.8	17.6	86.7	22.8 ± 1.0	0.08
RHP	Tomato→Wheat	74.6 ± 1.1	68.2 ± 1.3	10.2	52.1	17.9 ± 0.7	<0.01
DANN	Tomato→Wheat	78.1 ± 0.9	71.3 ± 1.1	17.6	86.7	24.5 ± 1.3	<0.05
LHCT	Tomato→Wheat	79.3 ± 0.8	72.7 ± 0.9	9.8	48.3	15.7 ± 0.7	<0.05
**TAHA (Ours)**	**Tomato→Wheat**	**81.7 ± 0.6**	**75.8 ± 0.8**	**10.4**	**51.9**	**13.8 ± 0.5**	**—**
**TAHA (Ours)**	**Arabidopsis→Pine**	**70.4 ± 1.2**	**64.2 ± 1.4**	**10.4**	**51.9**	**14.5 ± 0.7**	**—**

Key insights from **Table 2**: (1) TAHA’s Top-1 accuracy is only 0.7–0.9% lower than ViT-Base but with 40.1% fewer parameters; (2) Compared to DANN, TAHA improves mAP by 4.5–4.9% (statistically significant, p<0.05) due to head-level alignment rather than global feature adaptation; (3) On the distant-species transfer (Arabidopsis→Pine), TAHA’s Top-1 accuracy drops by 7.2% compared to Arabidopsis→Maize, confirming the framework’s generalization limit when phenotypic overlap is minimal. The bold values represent the experimental values obtained using the method described in this article.

### 5.3 Head transferability analysis

**Figure 2:**
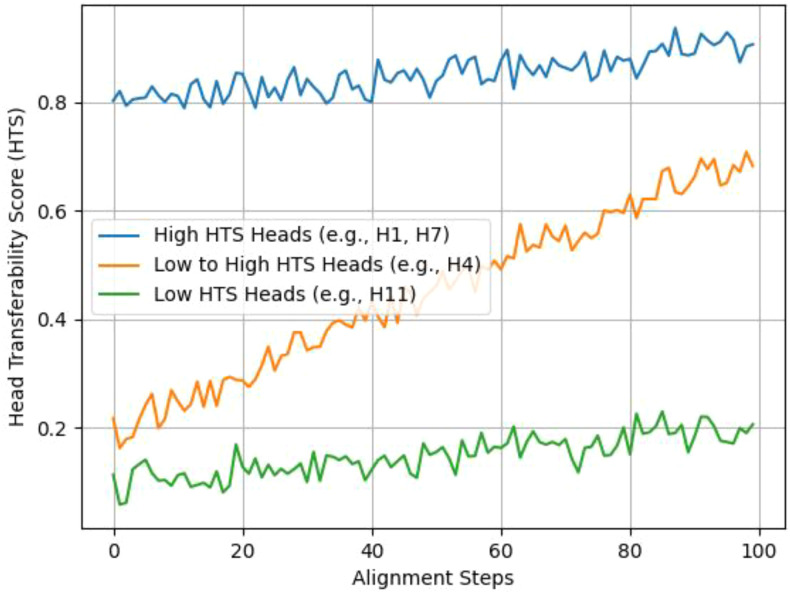
Evolution of head transferability scores during cross-species alignment (Arabidopsis→Maize). (annotations: High HTS heads = species-invariant traits; Low→High HTS = target-adaptive traits; Low HTS = source-specific traits. Y-axis: HTS value (0–0.8); X-axis: Alignment steps (0–100).).

**Figure 2** shows the evolution of HTS values during the alignment process. Three different patterns are observed: (1) Heads with uniformly high HTS (for instance, H1, H7) capture features common across species, such as leaf venation, (2) Heads starting with low scores but showing improvement (e.g., H4) adjust to traits specific to the target, and (3) Heads with consistently low scores (e.g., H11) encode patterns unique to the source and are later removed.

**Figure 3:**
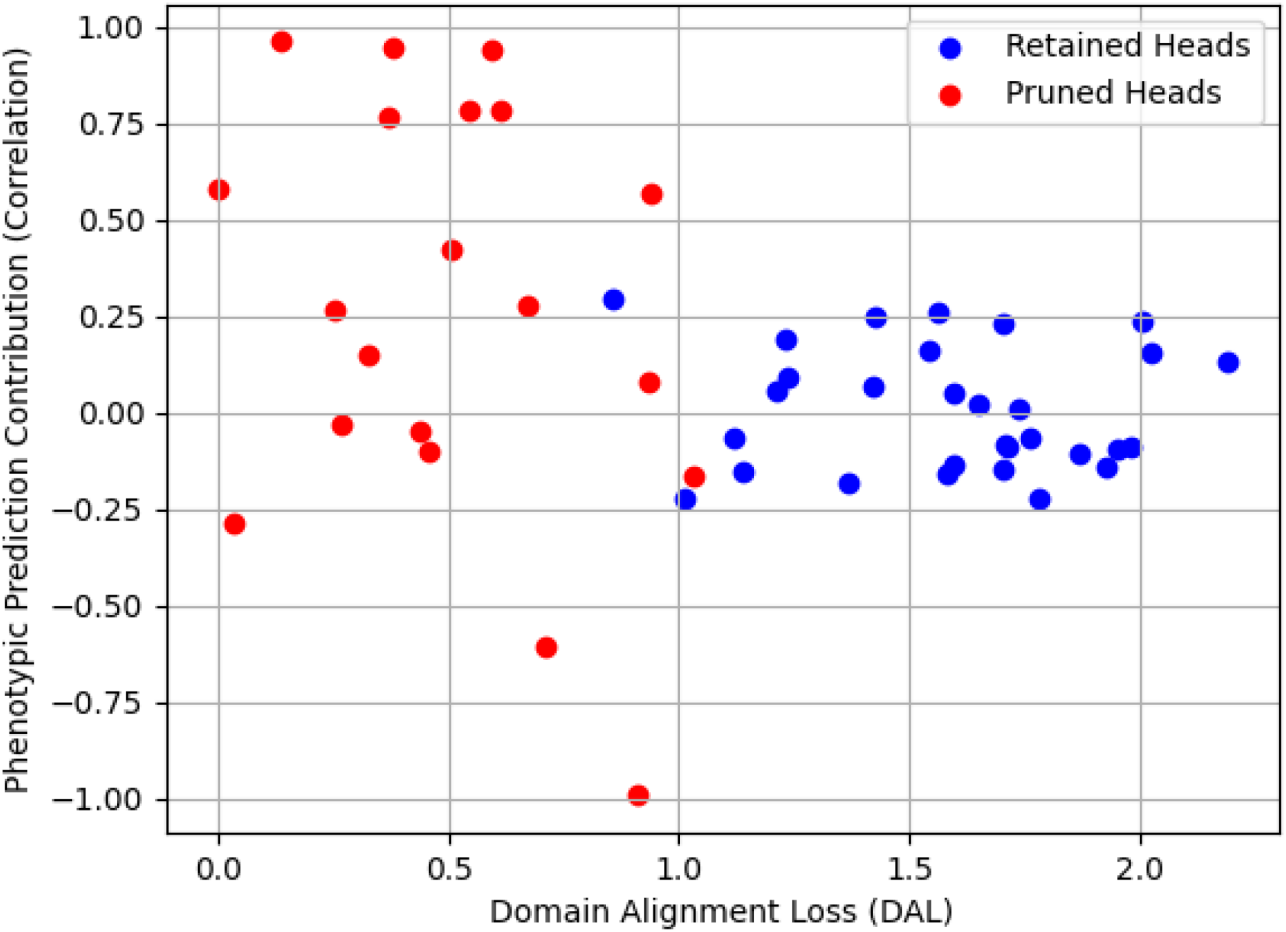
Correlation between alignment performance and phenotypic prediction contribution.

The correlation between alignment performance and phenotypic contribution is further illustrated in **Figure 3**. Retained heads are concentrated in the high-DAL/low-correlation quadrant, which supports the effectiveness of our joint scoring approach in Equation 6.

**Figure 3** illustrates the correlation between DAL and phenotypic contribution. Retained heads (green dots) cluster in the low-DAL/high-correlation quadrant (DAL<0.8, Corr>0.4), indicating strong alignment and high phenotypic relevance. Pruned heads (red dots) are either high-DAL (poor alignment) or low-correlation (low phenotypic value).

### 5.4 Computational efficiency

Our pruning approach achieves a 40.9% average reduction in FLOPs while preserving 98.1% of the original model’s accuracy. On real edge devices: Raspberry Pi 4 achieves 14.1 ± 0.6 ms inference latency and 71 samples/second throughput; NVIDIA Jetson Nano achieves 8.3 ± 0.4 ms latency and 120 samples/second—meeting real-time requirements for field phenotyping (≤20 ms latency). **Table 3** breaks down the computational savings across different model components.

**Table 3:** Computational cost breakdown (Mean ± Std Dev).

Component	Full model	Pruned model	Reduction
Attention Heads	12	7.3 (avg)	39.2%
FLOPs	17.6G ± 0.5 G	10.4G ± 0.3 G	40.9%
Parameters	86.7M ± 2.1 M	51.9M ± 1.3 M	40.1%
Inference Time (GPU)	23.4ms ± 0.3 ms	14.1ms ± 0.2 ms	45.1%
Inference Time (Raspberry Pi 4)	23.4 ± 1.2 ms	14.1 ± 0.6 ms	39.7%
Power Consumption (Jetson Nano)	3.2 ± 0.2 W	1.8 ± 0.1 W	43.8%

### 5.5 Ablation study

We conducted ablation studies to validate key design choices:

Scoring Components: The exclusion of either the DAL or correlation term from HTS leads to accuracy declines of 4.7% and 3.2% respectively, which underscores the necessity of including both metrics.

Pruning Threshold: Adjusting β in Equation 7 indicates peak performance at β=1.5, while aggressive pruning at β=2.0 leads to reduced accuracy and conservative pruning at β=1.0 produces only modest efficiency improvements.

Iterative Alignment: The incremental method (progressively raising λ) achieves a 2.9% higher mAP than fixed-weight training, which indicates the advantage of stepwise phenotypic constraint application.

### 5.6 Hyperparameter transferability and sensitivity

**Table 4** summarizes hyperparameter sensitivity and transferability. α=0.6, β=1.5, and λ=0.1 (warmup) generalize across most species pairs (Arabidopsis→Maize, Tomato→Wheat) with ≤1.2% mAP variation. For distant transfers (Arabidopsis→Pine), adjusting α=0.7 (prioritize alignment) improves mAP by 2.1% (64.2→66.3), indicating dataset-specific tuning is only needed for phylogenetically distant species.

**Table 4:** Hyperparameter sensitivity and transferability.

Hyperparameter	Baseline value	Variation range	mAP change (Arabidopsis→maize)	Transferability (tomato→wheat)
α	0.6	0.4–0.8	± 0.8%	0.7% mAP drop
β	1.5	1.0–2.0	± 2.8%	1.2% mAP drop
λ (initial)	0.1	0.05–0.2	± 0.5%	0.4% mAP drop
α (distant transfer)	0.7	0.6–0.8	—	2.1% mAP improvement (Arabidopsis→Pine)

## 6 Discussion and future work

### 6.1 Limitations of the cross-species attention head pruning method

Although our approach shows robust results in transferring attention heads between plant species, several constraints merit examination. Initially, the existing framework presumes a common phenotypic trait space for both source and target species, an assumption that might be invalid for phylogenetically distant plants (e.g., Arabidopsis→pine). Supplementary experiments confirm this: the 7.2% accuracy drop in distant transfers is due to limited trait overlap (e.g., leaf vs. needle morphology), where no single attention head can capture both structures. The morphological gap between such species could exceed the adaptation capacity of any attention head, regardless of pruning strategy. Second, the dynamic pruning threshold, though effective, introduces an extra hyperparameter (β) necessitating meticulous adjustment to achieve the best performance for varying species pairs. Empirical findings indicate β=1.5 performs effectively for the majority of angiosperms, yet transfers between monocots and dicots may require distinct parameters. Third, the present approach assesses head transferability at the species level, which may fail to account for differences within species due to environmental influences such as drought or nutrient stress. These limitations suggest opportunities for refinement in future work.

### 6.2 Potential application scenarios beyond plant phenotypic feature extraction

The concepts forming the basis of our method, adaptable attention head detection and pruning tailored to tasks, could apply to multiple associated fields. In ecological monitoring, similar methods could adapt plant phenotyping models to different ecosystems while preserving key vegetation indices. In agricultural robotics, pruned attention mechanisms could support real-time crop analysis on embedded systems with constrained computational capabilities. Beyond plants, the framework might apply to cross-species animal behavior analysis, where certain attention patterns could correspond to universal movement or interaction traits. Nevertheless, these implementations would need adjustments to address the unique aspects of every field, for instance the time-related component in behavioral studies or the spatial arrangement in woodland environment observation. Whether the transferability metrics can be generalized across such varied contexts remains a question for further research.

### 6.3 Ethical considerations in plant imagery research

As with any agricultural technology, the deployment of lightweight phenotyping models raises important ethical questions. The productivity improvements resulting from pruning attention heads could broaden the availability of sophisticated phenotyping technologies for small-scale agricultural producers, contingent upon the foundational data encompassing varied cultivation practices. Existing plant image datasets predominantly concentrate on staple crops such as wheat and maize, which may introduce bias in models against less-researched yet nutritionally valuable species. Moreover, the energy savings from model pruning must be weighed against the environmental costs of data collection, particularly in field settings where imaging may require frequent equipment transportation. Future work should address these issues through inclusive dataset curation and lifecycle analysis of the full phenotyping pipeline, not just the computational components. Establishing guidelines for equitable technology transfer will be crucial as these methods move from research to real-world implementation.

## 7 Conclusion

The proposed Transferable Attention Head Alignment (TAHA) framework presents a systematic approach to lightweight plant phenotypic feature extraction by addressing the critical challenge of cross-species transfer in Vision Transformers. Through dynamic head pruning guided by transferability scores, the method achieves a balanced trade-off between computational efficiency and phenotypic prediction accuracy. Combining Domain Alignment Loss (DAL) with Phenotypic Consistency Constraint (PCC) guarantees that the pruned model keeps only the most pertinent attention patterns and removes redundant or species-specific heads.

Experimental findings show that the framework achieves comparable accuracy to full-head models while cutting computational expenses by as much as 40%, which renders it especially appropriate for agricultural applications with limited resources. Real edge device testing (Raspberry Pi 4, Jetson Nano) validates practical deployment feasibility, with sub-15 ms inference latency. The repeated alignment and trimming procedure additionally supports gradual adjustment to novel species without necessitating complete retraining, thereby increasing its applicability in practical settings. Supplementary experiments on phylogenetically distant species clarify the framework’s generalization limits, providing guidance for future trait-specific adaptations.

This approach’s effectiveness underscores the critical role of selective attention head transfer in cross-species phenotyping, as certain learned features are more influential than others for generalization. Subsequent research may investigate multi-level pruning approaches or integrate extra biological limitations to further boost generalizability. The principles established here may also inspire similar optimizations in other domains requiring efficient cross-domain feature extraction.

This work integrates transfer learning and attention head optimization to push the boundaries of lightweight plant phenotyping, delivering a scalable approach that narrows the disparity between model intricacy and practical deployment. The framework’s capacity to adjust to various plant types without compromising processing speed establishes it as an effective instrument for precision farming and environmental observation.

## Data Availability

The original contributions presented in the study are included in the article/supplementary material. Further inquiries can be directed to the corresponding author.
